# Factors related with symptom duration until diagnosis and treatment of symptomatic colorectal cancer

**DOI:** 10.1186/1471-2407-13-87

**Published:** 2013-02-23

**Authors:** Magdalena Esteva, Alfonso Leiva, María Ramos, Salvador Pita-Fernández, Luis González-Luján, Montse Casamitjana, María A Sánchez, Sonia Pértega-Díaz, Amador Ruiz, Paloma Gonzalez-Santamaría, María Martín-Rabadán, Ana M Costa-Alcaraz, Alejandro Espí, Francesc Macià, Josep M Segura, Sergio Lafita, Francisco Arnal-Monreal, Isabel Amengual, Marta M Boscá-Watts, Angels Hospital, Hermini Manzano, Rosa Magallón

**Affiliations:** 1 Unit of Research, Majorca Department of Primary Health Care, Balearic Institute of Health, Reina Esclaramunda 9, 07003, Palma de Mallorca, Spain; 2 Department of Public Health, Balearic Department of Health, Majorca Cancer Registry, C/ Jesus n 33, 07001, Palma de Mallorca, Spain; 3 Clinical Epidemiology and Biostatistics Unit, A Coruña University, Complexo Hospitalario Universitario A Coruña, Xubias de Arriba, 84, Hotel de los pacientes 7ª planta, 15006, A Coruña, Spain; 4 Serreria II Primary Care Centre, Valencia Institute of Health, C/ Pedro de Valencia 28, 46022, Valencia, Spain; 5 Health Consorcium of Barcelona, Parc Sanitari Pere Virgili - Edifici Mestral, Esteve Terrades, 30, 08023, Barcelona, Spain; 6 Canal Imperial Primary Care Centre, Paseo Colon 4, 50006, Zaragoza, Spain; 7 Can Misses Primary Care Centre, Primary Health Care Eivissa Department, Avinguda de la Pau s/n, 07800, Eivissa, Spain; 8 Nazareth Primary Care Centre, Parque Nazareth 16, 46024, Valencia, Spain; 9 Department of Gastroenterology, University Clinic Hospital of Valencia, Avenida Blasco Ibañez 17, 46010, Valencia, Spain; 10 Evaluation and Clinical Epidemiology Department, Hospital del Mar, Passeig Marítim 25-29, 08003, Barcelona, Spain; 11 Lluis Saye Primary Care Centre, C/ Torres i Amat 8, 08001, Barcelona, Spain; 12 Pirinees High Resolution Hospital, Calzada de Rapit s/n, 22700, Jaca, Spain; 13 Department of Anatomical Pathology, A Coruña University, Complexo Hospitalario Universitario A Coruña, Xubias de Arriba, 84, Hotel de los pacientes 7ª planta, 15006, A Coruña, Spain; 14 Department of Anatomical Pathology, Hospital Universitario Son Espases, Carretera Valldemosa, Palma, Spain; 15 Department of Oncology, Hospital Universitario Son Espases, Carretera Valldemosa, Palma, Spain; 16 Primary Care Research Unit, Arrabal Health Centre, Gracia Gazulla, 50015, Zaragoza, Spain

**Keywords:** Colorectal cancer, Early diagnosis, Primary health care, Delay

## Abstract

**Background:**

Colorectal cancer (CRC) survival depends mostly on stage at the time of diagnosis. However, symptom duration at diagnosis or treatment have also been considered as predictors of stage and survival. This study was designed to: 1) establish the distinct time-symptom duration intervals; 2) identify factors associated with symptom duration until diagnosis and treatment.

**Methods:**

This is a cross-sectional study of all incident cases of symptomatic CRC during 2006–2009 (795 incident cases) in 5 Spanish regions. Data were obtained from patients’ interviews and reviews of primary care and hospital clinical records. Measurements: CRC symptoms, symptom perception, trust in the general practitioner (GP), primary care and hospital examinations/visits before diagnosis, type of referral and tumor characteristics at diagnosis. Symptom Diagnosis Interval (SDI) was calculated as time from first CRC symptoms to date of diagnosis. Symptom Treatment Interval (STI) was defined as time from first CRC symptoms until start of treatment. Nonparametric tests were used to compare SDI and STI according to different variables.

**Results:**

Symptom to diagnosis interval for CRC was 128 days and symptom treatment interval was 155. No statistically significant differences were observed between colon and rectum cancers. Women experienced longer intervals than men. Symptom presentation such as vomiting or abdominal pain and the presence of obstruction led to shorter diagnostic or treatment intervals. Time elapsed was also shorter in those patients that perceived their first symptom/s as serious, disclosed it to their acquaintances, contacted emergencies services or had trust in their GPs. Primary care and hospital doctor examinations and investigations appeared to be related to time elapsed to diagnosis or treatment.

**Conclusions:**

Results show that gender, symptom perception and help-seeking behaviour are the main patient factors related to interval duration. Health service performance also has a very important role in symptom to diagnosis and treatment interval. If time to diagnosis is to be reduced, interventions and guidelines must be developed to ensure appropriate examination and diagnosis during both primary and hospital care.

## Background

Colorectal cancer (CRC) is the second most frequent cancer and the second leading cause of cancer death in Europe [1]. CRC survival rates across the continent have increased mainly due to improvements in diagnosis and treatment with a mean survival rate of 56.2% at five years according to European cancer registries [2]. Survival depends mostly on stage at the time of diagnosis [3]. However, late diagnosis or treatment have also been considered as predictors of stage and survival, although these results are controversial [4-6].

Factors associated with the time interval between symptom onset and diagnosis or treatment are not sufficiently understood, regardless of whether they are related to the patient, the GP or the hospital setting. Theoretical frameworks used in cancer diagnosis research conceptualized time intervals occurring between phases of decision-making. [7,8]. These early models, recently refined by Walters et al. [9], included four intervals: ‘appraisal’, ‘help-seeking’ ‘diagnostic’ and ‘pre-treatment’, in an attempt to represent the processes occurring during each. In fact, the CRC diagnosis process is complex and difficulties may arise at any point along the clinical pathway. Firstly, the patient and his/her social context response, when faced with symptom onset, could determine time elapsed before seeking help [10]. In this sense, the severity value given to a symptom, and the attitudes when dealing with a possible cancer, can lead to either early help-seeking or waiting for the symptoms to clear up [11]. Moreover, in most cases, the tumor appears with very common and non-specific symptoms, and general practitioners (GPs) and secondary care specialists are required to differentiate between patients whose symptoms may be due to cancer and others whose symptoms are attributable to benign, transitory illness [12-14].

While there are recent rigorous studies on time duration to cancer diagnosis or treatment, much of the relevant literature is old and presents methodological issues that need to be taken into account such as different ways of measuring symptom duration and accuracy in recording symptom presentation [4,5].

Care continuity problems in CRC diagnosis have been highlighted in several countries, and health authorities advocate reduction in the time interval between suspicion of CRC and diagnosis or treatment. Various initiatives have been set up to identify those patients with symptoms related to CRC that suggest a need for prompt investigation [15,16].

In this study we try to address several issues. Firstly, to identify the delays that may occur at various stages along the clinical pathway in accordance with theoretical models, the factors associated with symptom duration and where there is greatest potential for improvement. Secondly, to overcome some of the problems identified in our reviews with a wider spectrum of patients and provide more accurate definitions of time intervals all over the diagnosis pathway and symptom presentation.

The aims of the study are: 1) to establish the distinct time-intervals to diagnosis and treatment; 2) to identify factors associated with late diagnosis and treatment.

## Methods

### Setting and study population

This paper reports findings from a larger study whose methods have been published elsewhere [17]. This is a cross-sectional study carried out in 5 Spanish regions. Briefly, subjects were consecutive incident cases of CRC (IDC9 153 and 154), verified histologically, between September 2006 and September 2008 and registered with a GP. Prevalent or recurrent cases were excluded as well as patients with multiple tumors and those diagnosed in private hospitals. For the present analysis, non-symptomatic patients were also excluded (screening detected cases, incidental findings).

Patients were identified at diagnosis through results of histological findings from endoscopy or surgery and contacted during the inpatient stage or oncology visit by their surgeon or oncologist who invited them to participate.

### Measurements

Data were obtained from patient interviews together with primary care and hospital clinical records review. Each of the patients was interviewed after diagnosis using a structured questionnaire during the inpatient stage, oncology visit or, if this were not possible, at home by a trained nurse or GP. Informed consent for study participation and access to medical records was obtained during the interview. In cases where the patient was deceased or could not be interviewed for other reasons, permission to review patient clinical records was obtained from a close family member. Details about measurements can be found in Additional file 1.

Interview measurements: Each patient was asked how long he/she had been feeling unwell and given a checklist to identify the type of symptom/s noted [18]. Symptoms spontaneously mentioned by the patient were considered the initial symptom/s for that patient and the date was recorded. Additional presenting symptoms were recorded. Other variables were social class [19] and demographic characteristics, perception of symptom seriousness, disclosure of symptoms, help-seeking action, doctor-patient relationship, and familial history of cancer. Primary care measurements were: date of first symptom and other symptoms, date of first contact with GP due to CRC symptoms, examinations and visits before diagnosis and their results, and date and information included in the referral. Hospital record measurements were: tumor data, date of first symptom and other symptoms, intestinal obstruction, date of first contact due to CRC symptoms, date of diagnosis, service attending the patient, examinations and visits before diagnosis and their results, and date and type of treatment.

### Symptom duration was divided into several intervals

Symptom Diagnosis Interval (SDI): from onset of first CRC symptoms to date of diagnosis.

Symptom Treatment Interval (STI): from onset of first CRC symptoms until start of treatment.

Patient Interval (PI): from onset of first CRC symptoms to date of first consultation with a physician (GP or hospital specialist).

Health Services Interval (HSI): Interval from 1st contact with a physician to diagnosis.

Treatment interval (TI): Interval from date of diagnosis to date of treatment.

In order to calculate time intervals, it was necessary to establish several definitions:

Date of onset of the first symptom/s was the one referred to by the patient. In the case of no interview, the date of 1st symptom recorded in the primary health care record or in the hospital record was used.

Date of diagnosis, i.e., date of first positive histology report. Date of treatment, i.e., date of surgery, date of preoperative or postoperative radiotherapy or chemotherapy or date of palliative treatment. For those patients who did not receive any treatment, the date of the visit closest to the decision not to treat the patient was recorded.

### Statistical analysis

The graphical analysis of interval times showed skewed distributions confirmed with the Kolmogorov-Smirnov test. Diagnosis or treatment intervals are presented as median and 25–75 percentiles. To assess the relationship between the different types of symptom interval duration and the observed variables, we used the Mann–Whitney U test for dichotomous variables and Kruskal-Wallis test for those variables with more than two categories. In order to avoid symptoms that could not be related to CRC, no more than a 24-month interval between symptom and diagnosis was accepted. Nineteen patients (0.3%) had a SDI of 24 months and twenty-one (0.7%) in the case of STI. All statistical analyses were performed using SPSS v.15.0.

This Study was approved by the Primary Health Care Committee of each health district and by the Ethical and Clinical Research Committee of each participating region.

## Results

A total of 950 incident colorectal cancer patients were recruited. For the present study, 155 asymptomatic patients were excluded; 94 (9.9%) screen detected and 61 (6.4%) incidental findings. Finally, 795 symptomatic cases were included; 481 (60.5%) were colon cases and 314 (39.5%) rectum. Hospital record information was available for all patients and primary care records for 780 patients (98.1%). In 66 (8.3%) patients the interview was not completed. The interview was completed during inpatient stay in 310 patients (44.7%), prior to surgery in 48 (6.9%), during outpatients visits in 267 (38.5%) and 69 (9.9%) at home.

Various time intervals are shown in Table 1. The median SDI and STI for CRC were 128 and 155 days respectively. Three months following symptom onset, four in ten patients are diagnosed and nearly three treated. No statistically significant differences were observed in SDI between colon and rectum, 125 (59–258) vs. 121 (53–256) respectively (p=0.51), or in STI 150 (80–280.5) vs. 153 (79–283), (p=0.08). Most of the treatment interval stemmed from health services time interval to diagnosis and less from the patient or interval from diagnosis to treatment.

**Table 1:** Distribution of delay intervals (in days)

	**Symptom diagnosis interval**	**Symptom treatment interval**	**Symptom-patient first contact with a doctor interval**	**Health services interval**	**Diagnosis to treatment interval**
	Median (P25-P75)	Median (P25-P75)	Median (P25-P75)	Median (P25-P75)	Median (P25-P75)
Colon & rectum	128 (57.5-257.5)	155 (84.0-283.5)	19 (3–83)	66 (25–159)	22 (8–37)
Colon	125 (59.0-258.0)	150 (80.0-280.5)	18 (3–74)	64 (23–164.5)	18 (4–33)
Rectum	121 (53.0-256.0)	153 (79.0-283.0)	20 (3–83.2)	62 (22–156)	22 (8–38)
	N(%)	N(%)	N(%)	N(%)	N(%)
Colon & Rectum					
< 1 month	110 (14.1)	59 (7.9)	483 (61.7)	245 (30.8)	501 (65.8)
1-3 months	190 (24.3)	152 (20.4)	139 (17.8)	235 (29.6)	234 (30.7)
3-6 months	207 (26.5)	215 (28.9)	80 (10.2)	149 (18.7)	16 (2.1)
6 month-1 year	165 (21.1)	184 (24.7)	35 (4.5)	109 (13.7)	9 (1.2)
> 1 year	109 (14.0)	134 (18.0)	46 (5.9)	57 (7.2)	1 (0.1)

Women present higher diagnostic time intervals than men. No significant differences in symptom duration intervals were found for age, level of education, social class or marital status. Finally, those patients with family history of cancer had longer diagnosis and treatment time intervals (Table 2).

**Table 2:** Socio-demographic characteristics

	**N %**	**Symptom diagnosis interval median (P25-P75) (days)**	**Symptom treatment interval median (P25-P75) (days)**
Gender			
Men	489 (62.7)	113 (51.0-246.0)	144 (84.0-273.7)
Women	291 (37.3)	153 (73.0-274.0)	175 (93.0-289.0)
P		<0.01	0.07
Age group			
<50	45 (5.7)	171.0 (127.2-246.2)	149.0 (104.0-214.0)
50-64	188 (24.2)	163.0 (87.5-295.5)	133.0 (60.5-254.5)
65-74	223 (28.1)	137.0 (83.0-255.2)	112.5 (49.0-224.7)
>=75	321 (41.3)	159.5 (84.0-326.2)	132.0 (62.5-289.5)
P		0.34	0.20
Marital Status			
Single	61 (8.4)	130 (48.0-342.0)	157.5(75.5-359.0)
Married	517 (71.1)	120 (57.0-245.5)	153.0 (89.0-272.0)
Widow/Separated/Divorced	149 (20.5)	154 (77.5-306.5)	178.0 (96.0-336.7)
P		0.09	0.40
Level of Education			
Illiterate-incomplete primary	106 (14.6)	127.0 (39.0-265.5)	151.0 (73.0-282.0)
Primary education	402 (55.3)	132.0 (66.0-270.0)	163.0 (93.2-295.7)
Secondary education	101 (13.9)	130.0 (70.5-214.5)	160.0 (88.5-264.5)
High School	72 (9.9)	122.0 (47.0-311.0)	155.0 (79.0-343.0)
University education	46 (6.3)	103.5 (34.5-232.2)	127.0 (55.5-260.0)
P		0.78	0.78
Social Class			
Class I & II	58 (9.0)	145.5 (67.7-267.5)	146.5 (80.5-313.5)
Class III	154 (23.8)	133.0 (63.0-320.5)	163.0 (89.5-354.0)
Class IVa	208 (32.2)	120.0 (58.5-223.5)	154.5 (93.5-255.0)
Class IVb	102 (15.8)	131.0 (66.5-244.5)	170.5 (92.2-296.2)
Class V	124 (19.2)	128.0 (48.5-229.0)	155.0 (77.5-257.0)
		0.86	0.73
History of cancer in family members and/or acquaintances’			
Yes	329 (45.3)	138.0 (70.0-276.0)	169.5 (96.2-313.7)
No	398 (54.7)	124.5 (56.0-227.0)	147.0 (78.5-259.5.2)
P		0.07	0.01

Concerning initial symptoms (Table 3), the presence of abdominal pain, vomiting and intestinal obstruction are associated with shorter time between symptom onset and diagnosis or treatment. There were no differences between SDI and STI with regard to other symptoms or the number of symptoms at presentation. Moreover, we observed a clear gradient in the duration of SDI depending on perceived symptom severity with shorter SDI and STI for those perceived as very serious. Therefore, the duration of intervals is related to patient attitude toward the symptom, that is, the interval duration diminishes when patients disclose the symptom to those close to them; when they do not wait for symptom clear up, or when they visit emergency services (either primary care or hospital). We found an association between trust in the GP and time to diagnosis or treatment.

**Table 3:** Relationship with presenting symptom, help-seeking behaviour and trust in their GP

	**N (%)**	**Symptom diagnostic interval**	**Symptom treatment interval**
		**median (P25-P75)**	**median (P25-P75)**
		**(days)**	**(days)**
Abdominal pain			
Yes	195 (27.3)	117.0 (41.0-220.0)	137.0 (58.0-252.0)
No	520 (72.7)	133.0 (66.0-280.0)	171.0 (96.7-314.0)
P		0.01	<0.001
Other pain			
Yes	50 (7.0)	122.5 (66.7-359.0)	159.5 (98.7-394.5)
No	665 (93.0)	130.0 (59.0-259.0)	159.0 (87.0-288.0)
P		0.70	0.52
Rectal Bleeding			
Yes	285 (39.9)	118.0 (51.5-260.0)	169.0 (89.0-298.0)
No	430 (60.1)	136.0 (63.0-263.0)	154.0 (85.5-282.5)
P		0.38	0.43
Changes in bowel habits			
Yes	273 (38.2)	128.0 (57.0-229.0)	152.0 (86.7-275.0)
No	442 (61.8)	130.5 (61.7-275.2)	162.0 (89.0-317.0)
P		0.28	0.28
Vomiting			
Yes	24 (3.4)	49.0 (10.2-134.7)	71.0 (13.0-154.0)
No	691 (96.6)	131.0 (63.0-264.5)	161.5 (91.0-291.2)
P		<0.001	<0.001
Tenesmus			
Yes	38 (5.3)	146.0 (51.0-272.0)	181.0 (93.5-325.5)
No	677 (94.7)	130.0 (60.0-262.2)	155.5 (87.0-286.2)
P		0.67	0.40
Loss of appetite			
Yes	49 (6.9)	133.0 (45.0-192.0)	135.0 (59.2-214.7)
No	666 (93.1)	130.0 (61.0-268.0)	159.0 (89.0-296.0)
P		0.24	0.10
Loss of weight			
Yes	57 (8.0)	121.0 (58.5-200.0)	154.0 (92.0-233.0)
No	658 (92.0)	130.0 (60.0-271.0)	159.0 (87.5-299.5)
P		0.31	0.36
Fatigue			
Yes	97 (13.6)	145.0 (72.0-266.0)	172.0 (93.0-295.0)
No	618 (86.4)	128.0 (56.0-261.0)	155.0 (86.7-287.5)
P		0.29	0.49
Anaemia			
Yes	51 (7.1)	133.0 (43.0-365.0)	158.0 (66.0-515.0)
No	664 (92.9)	130.0 (61.0-256.2)	159.0 (90.0-282.0)
P		0.66	0.44
Obstruction			
Yes	106 (13.6)	88.0 (26.0-165.5)	86.0 (20.5-159.0)
No	675 (86.4)	131.0 (64.0-269.0)	169.0 (94.0-304.5)
P		<0.001	<0.001
Nº of initial symptoms			
1	417 (58.3)	131.0 (62.5-271.5)	160.5 (91.7-289.7)
2	160 (22.4)	124.0 (59.5-300.2)	158.5 (84.0-330.2)
3	72 (10.1)	143.5 (70.2-246.5)	161.0 (100.0-264.0)
> 3	66 (9.2)	89.0 (40.5-182.0)	117.5 (60.7-223.0)
P		0.12	0.21
Perception of seriousness			
Not serious	467 (65.6)	142.0 (67.5-285.5)	171.0 (94.0-316.5)
Quite serious	162 (22.8)	117.0 (61.7-263.2)	153.0 (91.0-296.5)
Very serious	48 (6.7)	83.0 (34.2-129.2)	119.0 (65.0-180.0)
Other	35 (4.9)	110.0 (43.0-229.0)	118.0 (68.0-247.0)
P		<0.001	<0.01
Symptom Disclosure			
Yes	87 (12.1)	126.0 (56.0-255.0)	155.0 (84.0-282.5)
No	634 (87.9)	152.5 (86.7-340.5)	173.0 (108.5-354.0)
P		0.04	0.06
Help Seeking behaviour			
Visited a doctor	494 (68.8)	123.5 (50.7-230.2)	153.0 (78.5-264.0)
Wait for clear up	184 (25.6)	167.5 (88.0-344.7)	187.5 (119.0-273.0)
Other	40 (5.6)	83.0 (33.7-200.0)	95.0 (45.2-232.0)
P		<0.001	<0.001
Health services first contact			
With my GP	530 (72.7)	147.5 (77.0-278.5)	174.0 (99.0-307.7)
Family Practice emergencies	37 (5.1)	85.5 (30.7-151.2)	114.0 (39.5-179.2)
Hospital emergencies	101 (13.9)	71.0 (22.0-169.7)	84.0 (36.0-204.0)
To my hospital doctor	27 (3.7)	146.0 (96.0-293.0)	190.5 (130.2-344.7)
To Private hospital	14 (1.9)	95.0 (67.7-344.7)	181.0 (113.0-32.0)
Other	20 (2.7)	66.0 (29.5-153.5)	111.0 (44.0-197.0)
P		<0.001	<0.001
Would you recommend your doctor?			
Yes without any doubt	412 (63.2)	129.0 (59.5-266.5)	156.0 (87.0-289.0)
Probably yes	171 (26.2)	128.0 (56.0-260.0)	159.0 (84.0-307.0)
Probably not	35 (5.4)	130.0 (88.0-213.0)	155.0 (105.0-268.5)
Absolutely not	34 (5.2)	224.0 (133.7-361.5)	228.0 (152.5-362.5)
P		0.02	0.11

With regard to GP performance (Table 4), some non-indicated investigations, such as abdominal transit and gastroscopy were prescribed very rarely: in only 6 and 16 patients, respectively. GPs requested 109 colonoscopies, 4 rectoscopies and 3 rectosigmoidoscopies. When general practitioners ask for diagnostic tests of blood, iron, or blood in the stools, the time to diagnosis or treatment increases. No relationship has been detected between these time intervals and lack of abdominal or rectal examination and other image investigations. Shorter SDI and SDT were observed when the general practitioner referral to hospital was urgent or when the GP mentioned suspected diagnosis in the referral. Greater duration to diagnosis was observed in those with an increasing number of visits to the GP for symptoms related to CRC and shorter duration for those frequently attended by their family or nursing practitioner in the previous year.

**Table 4:** Relationship of GP performance with delay

	**N (%)**	**Symptom diagnosis interval**	**Symptom treatment interval**
		**median (p25-p75)**	**median (p25-p75)**
		**(days)**	**(days)**
Abdominal exploration			
Yes	244 (30.7)	125.0 (67.0-223.0)	155.5 (93.2-259.2)
No	550 (69.3)	149.0 (70.0-280.0)	181.0 (93.7-315.2)
P		0.15	0.09
Rectal examination			
Yes	180 (32.8)	129.0 (72.0-250.0)	166.0 (98.2-279.0)
No	369 (67.2)	142.0 (69.5-261.0)	168.0 (91.7-291.0)
P		0.69	0.89
Faecal occult blood			
Yes	112 (20.3)	176.0 (89.5-292.0)	202.5 (118.7-324.5)
No	440 (79.7)	128.0 (62.0-248.0)	159.5 (89.7-278.2)
P		0.01	0.01
Blood test			
Yes	314 (56.9)	165.0 (88.7-300.7)	188.0 (114.0-323.0)
No	238 (43.1)	103.5 (44.7-223.2)	132.0 (77.5-251.5)
P		<0.001	<0.001
Iron deficiency investigation			
Yes	117 (21.4)	176.0 (100.0-328.0)	213.0 (116.5-364.0)
No	431 (78.6)	127.0 (63.0-251.5)	160.0 (89.5-272.5)
P		<0.01	<0.01
Abdominal XR			
Yes	22 (4.0)	180.5 (76.0-257.0)	187.0 (69.0-269.0)
No	528 (96.0)	132.5 (68.5-258.0)	166.0 (94.0-290.0)
P		0.36	0.90
Abdominal Echography			
Yes	52 (9.5)	145.5 (73.0-256.0)	165.5 (86.2-261.7)
No	498 (90.5)	136.0 (69.0-258.0)	167.5 (94.0-289.5)
P		0.74	0.74
Barium enema			
Yes	38 (6.9)	132.0 (60.0-272.2)	163.0 (96.0-306.0)
No	512 (64.4)	137.5 (70.0-257.7)	167.0 (93.0-287.0)
P		0.71	0.97
Colonoscopy			
Yes	109 (19.8)	165.0 (82.5-291.0)	195.0 (132.5-322.0)
No	441 (80.2)	129.5 (64.0-251.0)	157.0 (89.5-282.0)
P		0.02	<0.01
First referral to hospital			
Gastroenterology	218 (58.6)	149.5 (77.2-291.2)	185.0 (114.0-317.0)
Surgery	36 (9.7)	153.5 (58.0-291.0)	181.0 (115.2-299.0)
Internal Medicine	13 (3.5)	150.0 (81.0-247.5)	162.5 (106.0-228.2)
Emergency service	89 (23.9)	125.0 (50.0-238.7)	158.0 (82.7-249.0)
Others	16 (4.3)	220.5 (117.5-351.7)	232.0 (138.0-327.0)
P		0.27	0.36
Type of referral			
Ordinary	126 (37.5)	181.0 (84.0-303.0)	195.0 (117.2-304.2)
Preferential	99 ( 29.5)	163.0 (85.0-294.0)	193.0 (117.2-317.7)
Urgent	111 (33.0)	111.5 (56.5-208.2)	152.0 (90.0-237.0)
P		<0.01	<0.01
Diagnosis suspicion			
Yes	184 (54.6)	129.0 (62.0-265.0)	166.0 (92.0-297.0)
No	153 (45.4)	172.5 (86.7-292.0)	195.0 (119.0-317.0)
P		0.06	0.03
CRC suspicion in referral letter			
Yes	71 (22.4)	112.0 (63.7-215.7)	153.0 (106.0-247.0)
No	246 (77.6)	166.0 (78.0-304.0)	191.0 (109.0-318.0)
P		0.04	0.03
Number of visits due to CRC symptoms			
0	142 (25.9)	08.0 (48.2-205.75)	143.0 (83.0-241.7)
1-2	211 (38.5)	124.5 (59.7-223.0)	153.0 (87.7-256.2)
3-5	138 (25.2)	163.0 (81.0-296.2)	183.0 (104.0-308.5)
>=6	57 (10.4)	264.0 (27.7-393.7)	279.5 (165.0-405.7)
P		<0.001	<0.001
Visits in last 12 months before diagnosis			
0	7 (1.3)	212.0 (20.0-662.0)	244.0 (57.0-185.0)
1-5	148 (27.0)	126.5 (70.5-229.5)	169.0 (94.0-259.5)
6-12	194 (35.3)	120.0 (60.0-222.0)	153.0 (84.0-255.0)
13-24	139 (25.3)	160.0 (80.0-214.0)	182.5 (108.2.0-358.5)
>=25	61 (11.1)	154.5 (75.2-244.0)	171.0 (92.0-296.0)
P		0.06	0.05

CRC: Colorectal Cancer.

Regarding secondary care of CRC cases (Table 5), we observed that the first contact is made mainly through the Emergency or Gastroenterology services. Sixty-three per cent of patients were referred by their GP and a quarter contacted hospitals by themselves. Shorter intervals are observed when the patient contacts the emergency service on his or her own initiative. Additionally, patients with abdominal or rectal examination, blood test, XT or CT, are diagnosed or treated earlier; in contrast with patients with MRI.

**Table 5:** Performance of hospital doctors and relationship with delay

	**N (%)**	**Symptom diagnosis**	**Symptom treatment interval**
		**median (p25-p75)**	**median (p25-p75)**
		**(days)**	**(days)**
Service of first consultation			
Gastroenterology	312 (39.5)	168.0 (90.0-317.5)	199 (124.0-352.0)
General Surgery	52 (6.6)	159.0 (83.5-349.0)	223 (128.0-382.5)
Internal Medicine	36 (4.6)	146.5 (70.7-315.5)	145.5 (90.0-334.0)
Emergency service	363 (46.6)	88.0 (33.5-186.5)	108.0 (52.0-207.5)
Other	26 (3.3)	149.0 (84.0-281.0)	176.0 (103.0-294.5)
P		<0.001	<0.001
Contact with hospital			
Patient own referral	178 (24.8)	84.0 (27.5-163.5)	111.0 (40.0-210.5)
GP or out of hours service	452 (63.0)	140.0 (70.0-271.0)	171.0 (97.0-306.0)
Referral other specialists	54 (7.5)	126.0 (75.7-352.5)	144.5 (101.0-364.2)
Other	37 (4.7)	109.0 (66.5-240.7)	127.5 (85.2-264.5)
P		<0.001	<0.001
Requested tests			
Abdominal examination			
Si	583 (73.6)	111.0 (49.0-238.0)	136.0 (71.0-268.0)
No	209 (26.4)	144.0 (78.2-293.7)	189.0 (111.2-329.5)
P		<0.01	<0.001
Rectal Examination			
Si	423 (53.3)	118.0 (49,0-224,0)	151.0 (75.0-274.0)
No	370 (46.7)	132.0 (68.5-284.5)	162.0 (92.0-315.0)
P		0.05	0.09
Faecal Blood Test			
Si	41 (5.2)	150.0 (72.5-269.5)	193.0 (101.0-362.0)
No	752 (94.8)	127.0 (55.0-253.0)	154.0 (84.0-281.0)
		0.68	0.28
Blood Test			
Si	573 (72.5)	116.0(49.0-238.0)	139.0 (72.0-262.0)
No	217 (27.5)	144.5(83.0-290.5)	189.0 (119.5-335.2)
P		<0.001	<0.001
Iron Investigation			
Si	227 (28.7)	137.5 (61.0-286.0)	166.0 (90.0-314.0)
No	563 (71.3)	125.0 (55.5-245.0)	154.0 (83.02-75.5)
P		0.23	0.36
Abdominal XR			
Si	274 (34.6)	98.0 (33.0-195.0)	110.0 (48.5-211.5)
No	519 (65.4)	142.0 (72.0-293.0)	177.0 (108.5-318.5)
P		<0.001	<0.001
Abdominal Echography			
Si	141 (17.8)	113.5 (59.0-241.0)	134.5 (89.2-259.0)
No	652 (82.2)	130.0 (53.0-256.0)	160.5 (83.7-287.5)
P		0.51	0.20
Barium Enema			
Yes	61 (7.7)	145.0 (76.0-276.0)	179.0 (77.0-343.0)
No	731 (92.3)	124.0 (56.5-251.0)	154.0 (84.0-281.25)
P		0.63	0.63
Barium transit			
Yes	11 (1.4)	193.0 (84.5-247.0)	233.0 (47.0-291.0)
No	782 (98.6)	125.5 (57.0-253.0)	154.0 (84.0-282.0)
P		0.54	0.56
Gastroscopy			
Yes	92 (11.6)	117.0 (72.0-280.0)	167.0 (92.0-321.0)
No	699 (88.4)	127.0 (53.0-250.5)	154.0 (83.5-275.5)
P		0.41	0.34
Rectoscopy			
Yes	35 (4.4)	182.0 (66.0-324.0)	204.0 (103.7-388.0)
No	755 (95.6)	125.5 (56.0-251.0)	154.0 (83.5-280.0)
P		0.19	0.06
Rectosigmoidoscopy			
Yes	18 (2.3)	83.0 (43.0-180.0)	116.5 (77.0-221.2)
No	772 (97.7)	128.0 (59.0-255.0)	155.0 (84.0-287.0)
P		0.30	0.46
CT Abdominal Scan			
Yes	345 (43.6)	112.0 (45.5-221.5)	128.0 (69.2-244.7)
No	447 (56.4)	142.0 (64.0-280.0)	180.0 (97.5-316.5)
P		0.01	<0.001
Abdominal MRI			
Yes	40 (5.1)	194.0 (84.0-403.0)	227.0 (112.5-451.0)
No	751 (94.9)	124.5 (56.0-251.0)	153.5 (83.0-276.7)
P		0.02	0.01
Diagnosis orientation after first Hospital contact			
Correct	386 (50.5)	113.5 (55.7-228.5)	146.5 (83.5-263.5)
Appropriate	334 (43.7)	140.0 (58.0-257.5)	158.0 (78.5-297.5)
Inappropriate	44 (5.8)	137.0 (82.0-256.2)	172.0 (111.5-298.2)
P		0.39	0.50

*XR* Radiography, *CT* computed tomography, *MRI* Magnetic Resonance Image.

## Discussion

This study has used a comprehensive approach in assessing the factors that underlie the social and clinical pathway to cancer diagnosis duration in CRC. Findings show, as in the review of Mitchell et al. [11], the complex relationship between symptom presentation and patient behaviour regarding perception and response to the symptoms. Moreover, our results add more information on the effect in time elapsed from symptom to diagnosis or treatment of factors such as gender, attitude towards symptoms, appropriate GP referral and hospital doctor performance.

First of all, after experiencing the first CRC symptoms, half of the patients have to wait at least 4 months until diagnosis. This finding is similar to that described by others [20-24]. Most notable is the fact that one third has to wait more than six months to be diagnosed. In accordance with other findings [20,25], no differences in distinct symptom duration intervals by cancer site were detected, although some studies reported longer time for rectum [26,27].

Women in our study are more likely to experience longer time to diagnosis than men. These results probably are related to gender differences in coping with symptoms and help-seeking behaviour [10,28-30]. Women push men to see a doctor when they have CRC symptoms while the women themselves wait for symptoms to clear up [8] because of fear of having cancer [31]. In fact, waiting for symptom clear-up was associated in our study with not being diagnosed or treated promptly.

There are no conclusive results on socioeconomic status and time elapsed to diagnosis and treatment. Some studies show longer symptom duration in those patients with lower social status [22,28,32,33] while our data, in accordance with others [34,35], could not confirm these differences. Longer lag time is also seen in those patients with history of cancer in family members, friends or colleagues. These factors have not been researched in depth with regard to CRC and delay [11]. While Ratcliffe et al. describe no differences in time intervals in those with or without family history of cancer [36], other authors have suggested that prior experience of the disease through relatives or friends could result in putting medical consultation off [10,37].

Regarding symptom at presentation, we confirmed that abdominal pain [38-40] and vomiting [32,39] lead to shorter interval duration. At the same time, as described by other authors [28,40], our results show that beliefs that the symptom is serious and symptom disclosure to friends and family are strongly associated with shorter lag times. Again, this could be related to gender, as women with CRC symptoms generally say nothing to their families until they have already visited a doctor [10].

Current practice by general practitioners may contribute to lengthen time to diagnosis. For instance, we observed that when the GP requests some tests to diagnose anemia, the diagnosis process is extended [41]. Unfortunately, there is no simple solution to this. When dealing with a symptom such as anemia, with low predictive value, doctors must confirm non-malignancy before filling outpatient services with mild pathologies [13,14]. Similarly, misinterpretation of a symptom may result in a greater number of visits to a doctor before referral to a specialist [32-34,39-43], and this is a variable associated in our study with longer time to diagnosis or treatment.

Furthermore, erroneous orientation can be the result of poor examination and misdiagnosis. Our results show that the GP only carried out physical examination of one in three patients; in fact, prior studies have pointed out insufficient physical examination by the GP, and no improvement has been observed over time [40-42]. Lack of physical examination and poor investigations by outpatient or emergency doctors seem to have more consequences than family doctor performance [32]. This could be partly due to the fact that a misdiagnosis in a primary care visit is less important because continuity of care is highly assured. A misdiagnosis in outpatient care would be more relevant because a further appointment is considerably more difficult to make.

On the other hand, the capacity of the GP to form a suspicion of CRC in the referral has been shown to be a key factor as it predicts less time to diagnosis. This may be because when the GP has a clear suspicion, the referral is made more promptly and the response of hospital doctors to this type of referral is faster [44,45].

### Strengths and limitations

In contrast with previous studies limited to the assessment of partial time intervals to diagnosis or treatment (patient or system time intervals), we have been able to show that these factors also affect total time to diagnosis or treatment. Our findings show a similar relationship for time to diagnosis and time to treatment with the various factors studied. This fact demonstrates that the variables considered in this study have great influence on the time to diagnosis and, as a matter of fact, duration between diagnosis and treatment is relatively less relevant (>65% of those diagnosed are treated within a month). The inclusion of a high number of hospitals and primary care centers could imply high heterogeneity in data acquisition, but at the same time represents a complete picture of patterns of care, preventing the selection bias that appears when only a single centre is included.

The major limitation of this study lies in the lack of a consistent methodology to precisely measure the date of symptom presentation. The date of onset of symptoms obtained from the patient interview has been questioned. According to some authors, studies fail to consider existing theories of symptom interpretation. The interpretation of bodily sensations as symptoms is embedded within a social and cultural context and respondents do not define time periods in the same way [46]. Therefore, this date may be quite precise for acute symptoms, such as rectal bleeding, but difficult to establish for more general symptoms such as fatigue [47]. However, other studies have shown good agreement when comparing information expressed by patients and doctors [18,48]. Retrospective review of clinical records presents the same type of problem because it comes from patient information provided to doctors. Furthermore, some of the symptoms described to doctors may go unrecorded, as highlighted by some authors [49,50]. Patient interviews conducted during pre or post first treatment could result in memory biases. The Aarhus statement will facilitate standardized and uniform definition of studies in this area [51]. Finally, a trusting relationship with the GP, recorded after diagnosis, could be spurious as, patient faith in their GP, could be related to lag time to diagnosis or treatment [52,53].

## Conclusions

In conclusion, a substantial proportion of CRC patients experience long diagnosis and treatment intervals with potential impact on psychological wellbeing, quality of life or even survival. Time taken to diagnosis and treatment of colorectal cancer appears to be subject to many previously suspected factors, symptom presentation, patient response to symptom, and general practice and secondary care performance factors.

### Implications for further research and policy development

Research is needed to develop interventions for use by primary and secondary care doctors to reduce diagnostic and treatment time intervals. Gender differences encountered in our population also deserve to be investigated more thoroughly. Further research into first-symptom presentation and concordance between patients and primary and hospital clinical records would give further insights into methodological issues in cancer delay studies.

The results of this work reveal the existence of areas for improvement in health care delivered to patients. Particularly important is to introduce changes to the existing guidelines whereby physical examination and laboratory and image investigations are carried out on all those who present with lower gastrointestinal symptoms independently of age, gender or symptom duration. This would also favour thorough information being included in the referral letter. If time to diagnosis is to be reduced, there must be development of interventions and guidelines to ensure appropriate diagnosis and examination during both primary and hospital care.

## Abbreviations

CRC: Colorectal cancer;CT: Computerized Tomography;GPs: General Practitioner;HSI: Health Services Interval;MRI: Magnetic Resonance Image;PI: Patient Interval;SDI: Symptom Diagnosis Interval;STI: Symptom treatment interval;TI: Treatment interval;XR: X Ray

## Competing interests

The authors declare that they have no competing interests.

## Authors’ contributions

ME, MR, AR, SPF, LGL, RM and MC, ACA contributed to the study subject and design. Data Acquisition: PGS, SP, SL, AC, AE, FAM, IA, MMR, AH, MAS, AR. Quality control and Algorithms: AL, MAS, SL, SP, JMS, ACA. Data analysis an interpretation ME, AL and MR, SPF, LGL, AE and MC, HM. Statistical analysis was done by ME and AL. Manuscript preparation: ME, MR, SPF, LGL, MAS and MC. All authors contributed to thoroughly review of the manuscript. All authors read and approved the final manuscript.

## Pre-publication history

The pre-publication history for this paper can be accessed here:

http://www.biomedcentral.com/1471-2407/13/87/prepub

## Supplementary Material

Additional file 1 Measurements. Click here for file
