# Supplementary material for: Factors related with symptom duration until diagnosis and treatment of symptomatic colorectal cancer
Source: BMC Cancer. 2013 Feb 23;13:87. doi: 10.1186/1471-2407-13-87 (PMC3598975; doi:10.1186/1471-2407-13-87)
Supplement: Additional file 1 — Measurements. [file 1471-2407-13-87-S1.docx]

**Additional file 1.- Measurements**

| **MEASUREMENTS FROM THE INTERVIEW**  **Demographic characteristic**: Marital status, level of education, social class, age (date of birth/date of interview).  **Family history of cancer:** Patients were asked if any severe family members or friends suffered cancer and type of cancer. A check list of family members were offered (parents, brothers/sisters, son/daughter, spouse, friends).    **Initial symptom/s:** Each patient was asked how long he/she had been feeling unwell and given a checklist of 22 symptoms to identify the type of symptom/s noted and avoid underreporting. Symptoms spontaneously mentioned by the patient were considered the initial symptom/s for that patient and the date was recorded.  For the analysis, some symptoms were grouped, that is, changes in bowel habits included diarrhoea, constipation and changes in the stools. Rectal pain and other pain were grouped in 'other pain'. Blood in the stools included rectal bleeding and black stools.  **Number of initial symptoms:** For the analysis, first symptoms were summed up.  **Additional presenting symptoms:** Patient was asked about further symptoms presented during the interval to diagnosis. In this case no date of presentation was recorded.  **Perception of symptom seriousness:** Patient was asked if the initial symptom/s was considered: Very serious, quite serious, not serious, other.  **Disclosure of symptoms.** Patient have to answer disclosure of symptoms to a list of family members and quittances (multiple response). Parents, brothers/sisters, son/daughter, spouse, friends, quittances.  For present analysis responses were aggregated: If patient disclosed his/her symptom to somebody or not.  **Help-seeking action.** Patient was asked what he/she did after onset of first symptom/s. Visit a doctor, wait for symptom to clear up, other.  **Date of** **health services first contact.**  **Health services first contact**: After feeling unwell, which doctor patient contacted**.** GP, family practice emergencies, hospital emergencies, private doctor or private hospital, other.  **Confidence in their GP:** patients were asked, would you recommend your doctor?. Yes without any doubt, probably yes, probably not, absolutely not. |
| --- |
| **HOSPITAL RECORD MEASUREMENTS:**  *Review of hospital records were done after interview. After identify date of diagnosis from pathology report, data manager proceeded to backward review in order to identify first contact related with CRC symptom/s.*  **Tumour localization, TNM.**  **Date of first symptom/s:** as text notes recorded by hospital doctors.  **Presence of intestinal obstruction:** as text notes recorded by hospital doctors.  **Date of first contact due to CRC symptoms:** as text notes recorded by hospital doctors.    **Date of diagnosis:** date of first positive histology report.    **First Service attending the patient:** Gastroenterology, general surgery, internal medicine, emergency service.    **Mode of patient contact with hospital:** Patient own referral, GP or out of hours service referral, other specialist referral.  **Examinations:** A checklist of possible physical examinations and investigations were recorded.  **Diagnosis orientation after 1rst hospital contact:** Correct (if doctor suggest presence of colorectal cancer), appropriate (if doctor do not mention CRC but request investigations or hospitalization), inappropriate (do not attain correct or appropriate orientations).  **Number of outpatient visits before diagnosis:** all outpatient visits related with CRC symptom/s were registered.  **Date and type of treatment,** i.e., date of surgery, date of preoperative or postoperative radiotherapy or chemotherapy or date of palliative treatment. For those patients who did not receive any treatment, the date of the visit closest to the decision not to treat the patient was recorded. |
| **PRIMARY CARE MEASUREMENTS**  *Review of primary care records were done after interview and hospital records review in order to have precise date of diagnosis. Primary care records were reviewed from date of diagnosis to 2 years backward in order to identify first contact for CRC symptoms.*  **Date of first symptom/s:** as text notes recorded by GP.  **Date of first contact with GP due to CRC symptoms.** as text notes recorded by GP  **Examinations:** A checklist of possible physical examinations and investigations were recorded.  **Number of visits before diagnosis due to CRC symptoms:** all visits to GP related with CRC after fist symptom/s were registered.    **Number of visits in the last 12 months before diagnosis:** all visits (GP and nurse) in the last 12 months were registered.  **Date and information included in the referral:** Date of referral was recorded and check if there was diagnosis orientation of cancer, CRC or no diagnosis orientation. |
